# Supplementary material for: Integrating multiple plant functional traits to predict ecosystem productivity
Source: Commun Biol. 2023 Mar 3;6:239. doi: 10.1038/s42003-023-04626-3 (PMC9984401; doi:10.1038/s42003-023-04626-3)
Supplement: Supplementary file 2 — SUPPLEMENTAL MATERIAL [file 42003_2023_4626_MOESM2_ESM.pdf]

Supporting Information

The files include:

1) Text S1 Detailed explanation of common nouns and abbreviations in this study

2) Text S2 Random forest models were used to distinguish the importance of different trait variables

3) Text S3 Plot settings and biomass calculation

4) Text S4 Determination of plant functional traits

5) Text S5 Soil sampling and measurements

6) Text S6 The underlying mechanism of different path causal hypotheses

7) Table S1 The basic information for 72 sites across China

8) Table S2 Percentage contributions (%) and loadings of 4 individual soil variables to the first principal components ($Soil_{PC1}$)

9) Figure S1 Relative importance of different trait variables from random forest models

10) Figure S2 Observed gross primary productivity (GPP) versus predicted GPP for yearly (a) and monthly (b).

11) Figure S3 Correlation matrix among traits

12) Supplementary References

13) Supplementary Figure 2-5

Text S1 Detailed explanation of common nouns and abbreviations in this study

Plant community traits: community-level traits, including quantitative traits ($Trait_{quantity}$) and efficiency traits ($Trait_{efficiency}$).

$Trait_{quantity}$: quantity traits, standardize traits on the unit land area and represent the capacity for resource uptake and carbon fixation. The quantity traits included in this study are total leaf area (m^2 m^{-2}), biomass (g m^{-2}), nitrogen (g m^{-2}), and phosphorus (g m^{-2}) content per unit land area.

$Trait_{efficiency}$: efficiency traits, community-weighted mean traits and represent the efficiency of ecosystem production. The efficiency traits included in this study are leaf area, leaf dry mass (g), specific leaf area (cm g^{-1}), and leaf nitrogen (mg/g) and phosphorus (mg/g) concentration.

GSL: growing season length, and represents the number of months when plant growth takes place.

GPP_{yearly}: annual gross primary productivity (g C m^{-2} $year^{-1}$), and represents the amount of carbon fixed during photosynthesis by all producers in the ecosystem.

$GPP_{monthly}$: average monthly gross primary productivity (g C m^{-2} $month^{-1}$), and calculated as annual gross primary productivity divided by growing season length.

LA: leaf area (cm^{-2}).

LM: leaf dry mass (g).

SLA: specific leaf area (cm g^{-1}).

LNC: leaf nitrogen concentration (mg/g).

LPC: leaf phosphorus concentration (mg/g).

LAI: leaf area index (m^2 m^{-2}), and represents the one-sided green leaf area per unit land area.

LMI: leaf mass index (g m^{-2}), and represents the total dry matter of leaves per unit land area.

MI_{gs}: moisture index of growing season and represents that portion of the total precipitation during the growing season used to satisfy plant (vegetation) needs.

T_{gs}: mean annual temperature of growing season (°C).

P_{gs}: mean annual precipitation of growing season (mm $year^{-1}$).

SEM: structural equation modeling.

IEA: independent effect analysis.

LOOIC: leave-one-out cross-validation information criterion and smaller value is indicative of a better fit of Bayesian model.

ELPD: expected log predictive density and larger value is indicative of a better fit of Bayesian

model.

Text S2 Random forest models were used to distinguish the importance of different trait variables and to make modeling predictions.

Different from multi-model inference using linear regression or regression tree analysis, random forest [13] analysis: 1) This algorithm can handle multicollinearity by constructing an ensemble of bagged trees among explanatory variables; 2) This algorithm can automatically detect the best fit; 3) It reduces overfitting in decision trees and helps to improve the accuracy; 4) Normalising of data is not required as it uses a rule-based approach [13,14]. Therefore, we additionally used random forest model to distinguish the relative importance of all explanatory (Figure S2) variables to GPP (variables that are not correlated with productivity are excluded first). Further, random forests are used for modeling and prediction (Figure S3). The *rfPermute* package [15] was used to build the random forest model and to evaluate the significance of each variable included in the random forest model.

Text S3 Plot settings and biomass calculation

Based on the vegetation distribution in China, we conducted a systematic survey of 72 typical natural ecosystems, including forests, grasslands, and desert scrubs, covering the whole China almost evenly over the past six years (2013-2019). The whole study area spans 50 degrees of longitude (78.46 ~ 128.89 °E) and 35 degrees of latitude (18.75 ~ 53.33 °N). The average annual minimum temperature is -4°C, maximum temperature is 22°C, and annual precipitation is 25mm and 1785mm. In order to reduce human interference, survey samples are generally set up in nature reserves or near long-term monitoring samples near ecological stations.

1) For forests, four primary quadrats (30 × 40 m) were established in representative communities of each site to survey tree layers. In each primary quadrat, two 5 × 5 m and four 1 × 1 m sub-quadrats were used to survey shrub and herb layers, respectively. Some critical plant community parameters were measured, i.e., species identity, species number, plant height, diameter at breast height (DBH) for all woody plants with DBH ≥ 1 cm (basal stem diameter for shrubs), and aboveground biomass for each herbaceous species. For trees, the biomass of each organism was calculated using an allometric equation based on tree DBH (cm) and height (m), and the tree biomass allometric equation comes from Luo [1] and Luo, et al. [2]; for shrubs, we calculated the biomass based on the basal diameter and height, the shrub biomass allometric equation comes from Xie, et al. [3]; for herbs, the harvested herbaceous biomass was oven-dried at 70 °C for 72 h and weighed. In addition, for the desert (shrub) ecosystem, we set 6 shrub plots of 10 m × 10 m and 8 herb plots of 1 m × 1 m in each sample site. The quadrats were investigated in the same forest shrub layer and herb layer, respectively.

Leaf samples from trees were collected from the top sunny branches of individuals with good growth (four replicates for each species in each site) using a hanging tower, artificial climbing, and a high branch shear. Healthy leaves of each plant were combined to create a single leaf sample per species per plot. For shrubs, healthy leaves from the top branches were collected and mixed in each plot for every species. For grass, healthy leaves collected from 5 to 30 individual plants were mixed for each species. For rare species, leaf samples were collected from 1 × 1 km around plots to ensure a minimum amount of sampling. For more details about forest surveys, please refer to the peer-reviewed papers by Niu, et al. [4] and Zhang, et al. [5].

2) For grasslands, we set up eight plots (1 m × 1 m) at each site to investigate plant community parameters (such as species name, number, height, coverage) and harvested aboveground biomass of each species. These samples were dried at 85 °C for 72 h and weighed. Then, healthy leaves of each species were collected from five to 30 individual plants as a mixed sampling. We collected leaf samples from areas of 1 × 1 km around the plots for some rare species. For more details on the setting of grassland sites, please refer to the peer-reviewed papers of Li, et al. [6] and Xu, et al. [7].

Text S4 Determination of plant functional traits

1) Leaf area, leaf mass and specific leaf area

As Liu, et al. [8] statements, six to ten randomly selected healthy leaves (more for small-leaved species) of each species were placed on a portable scanner (Cano Scan LIDE 110, Japan) and flattened with a transparent plastic plate to ensure that the leaves were fully stretched. ImageJ software was used to obtain leaf area values. The scanned leaves were then dried in an oven at 60 °C for 48 h and weighed. SLA was calculated by dividing leaf area (LA, cm^2) by leaf dry mass (LM, g), as described in Eq. (1).

$$SLA = LA/LM \qquad (1)$$

2) Leaf nutrient (nitrogen and phosphorus) concentration

We used the agate mortar grinder to crush and grind all the dried samples, and the leaf nitrogen concentration was measured using an elemental analyzer (Vario Max C*N* Element Analyser; Elementar). The samples that were used for measuring P were acidified with 68% HNO3 [9], and then digested using a microwave digestion system. Finally, the P content was determined with an inductively coupled plasma optical emission spectrometer. More detailed information about sampling and measurements of these traits can be found in other peer-reviewed papers by this team [5,10-12].

Text S5 Soil sampling and measurements

Five soil samples of 0–10 cm depth were extracted from each of eight randomly placed 1 m × 1 m quadrats in each 1 km × 1 km sampling area using a soil sampler, and were air-dried after being sieved (2 mm mesh). All visible roots and organic debris were removed by hand. Samples were ground to a fine powder using a ball mill (MM400 Ball Mill, Retsch, Germany) and an agate mortar grinder (RM200, Retsch, Haan, Germany). Total soil carbon and nitrogen content were measured using an elemental analyzer (Vario MAX CN Elemental Analyzer, Elemental, Hanau, Germany). Total soil phosphorus content were determined using an inductively coupled plasma optical emission spectrometer (Optima 5300 DV; Perkin Elmer).

Text S6 The underlying mechanism of different path causal hypotheses

Firstly, environmental factors not only act as an energy input to power the ecosystem's carbon uptake, but also regulate the carbon distribution of vegetation, which directly affects ecosystem productivity [13]; secondly, $Trait_{capacity}$ (i.e. plant community traits) which standardize traits on the unit land area, represents the capacity of ecosystems carbon uptake [11]. Furthermore, Finally, the length of the growing season determines the effective period of carbon absorption in the ecosystem, and therefore has a positive influence on productivity [14]. In addition, traits represent the acclimatization and adaptation of plant communities to environments [15,16], so no matter how environmental factors change over time and space, they first affect the attributes of plant communities [15] and further influence ecosystem functions through changes in attributes [15,16]; in other words, due to the covariation of environmental factors and traits [17-19], a large part of the effect of environmental factors on productivity is supposed to be mediated by traits [20].

Table S1 The basic information for 72 sites across China

Site	Latitude (°N)	Longitude (°E)	Altitude (m)	MAT[‡] (ºC)	MAP (mm $year^{-1}$)
Jianfeng	18.74	108.86	860	20.70	1460
Xishuangbanna	21.60	101.58	785	22.20	1269
Dinghu	23.17	112.54	75	22.00	1942
Ailao	24.53	101.02	2505	12.90	1580
Jiulian	24.57	114.44	423	18.90	1690
Huanjiang	24.74	108.33	291	20.10	1687
Tianlong	26.25	105.76	1362	15.00	1184
Huanglian	28.19	106.80	1016	14.90	1061
Gutian	29.14	118.40	182	18.20	1517
Tiantong	29.80	121.78	73	16.50	1527
Jinyun	29.84	106.39	859	15.90	1463
Huping	30.11	110.80	1989	8.90	1852
Dujiangyan	31.11	103.56	1625	11.80	1006
Shennongjia	31.32	110.49	1402	10.10	1343
Taibai	33.26	107.36	934	13.20	1090
Taishan	36.22	116.31	53	14.10	591
Taiyue	36.68	112.10	1647	5.90	674
Dangling	39.97	115.48	891	7.70	485
Wuling	40.63	117.45	864	7.20	596
Qingyuan	42.02	124.94	541	5.50	857
Changbai	42.40	128.09	764	4.00	808
Longwan	42.62	126.26	318	6.20	640
Jiaohe	43.51	127.38	336	5.50	590
Maoer	45.30	127.55	338	3.40	806
Liangshui	47.18	128.89	366	1.80	684
Genhe	50.79	121.55	715	-2.00	395
Huzhong	51.76	123.29	812	-3.80	442
Mohe	53.33	121.15	600	-2.50	390
LP[†]01	36.29	113.36	819	10.50	475
LP 02	35.99	112.29	934	10.50	595
LP 03	35.99	111.64	762	11.40	555
LP 04	36.07	110.18	891	11.20	495
LP 05	36.74	109.24	1213	9.80	466
LP 06	36.93	107.92	1523	8.70	400
LP 07	37.58	107.19	1570	8.10	327

LP 08	37.42	105.78	1395	10.00	215
LP 09	37.44	104.92	1373	9.40	174
LP 10	37.46	104.44	1729	7.70	189
MP 01	44.59	123.51	140	6.40	413
MP 02	44.52	121.04	272	6.80	400
MP 03	45.11	120.33	680	3.60	379
MP 04	44.77	118.36	1017	1.80	321
MP 05	44.26	116.52	1113	2.40	324
MP 06	43.55	116.67	1273	2.30	307
MP 07	44.51	117.68	1188	2.30	317
MP 08	44.01	114.89	1104	3.10	200
MP 09	43.84	113.50	1018	4.20	189
MP 10	43.63	112.15	953	5.40	139
TP 01	31.46	95.45	4276	1.00	350
TP 02	31.85	93.53	4615	-0.60	370
TP 03	31.64	92.01	4588	-0.30	292
TP 04	31.38	90.74	4594	-0.10	359
TP 05	31.54	89.72	4599	0.10	186
TP 06	31.87	87.82	4571	-0.30	170
TP 07	31.92	85.84	4948	-2.50	242
TP 08	32.41	83.34	4599	-1.40	115
TP 09	32.30	81.23	4565	-3.20	53
TP 10	32.48	80.15	4565	-2.90	79
Maoxian	31.69	103.90	1814	9.80	717
Hongyuan	32.82	102.59	3448	2.80	559
Qiemo	36.94	82.40	1619	11.60	52
Cele	37.02	80.72	1308	12.50	32
Yecheng	37.46	78.47	1327	12.10	57
Linze	39.40	100.12	1392	9.20	92
Aksu	40.38	81.18	957	12.30	50
Shaya	40.80	82.09	920	12.20	43
Mulei	44.73	89.93	457	9.00	156
Kelamayi	45.29	85.02	217	10.60	116
Fuhai	46.22	90.07	946	5.00	136
Hulunbeier	49.35	120.12	676	-0.60	408
Moerdaoga	51.31	120.19	741	-2.40	430
Heishantou	50.17	119.39	531	-0.30	327

† LP, loess plateau; MP, Mongolian plateau; TP, Tibetan plateau;

‡ MAT, mean annual temperature; MAP, mean annual precipitation.

Table S2 Percentage contributions (%) and loadings of 4 individual soil variables to the first principal components (Soil$_{PC1}$)

Variables	Soil$_{PC1}$ [†]	
	Loading	Percent (%)
Soil_C(%)	0.94	31
Soil_N(%)	0.94	31
Soil_P(%)	0.70	23
Soil_pH	– 0.44	15

[†] The first principal components explained 61% of the variation in soil (only the first principal components eigenvalue is greater than one).

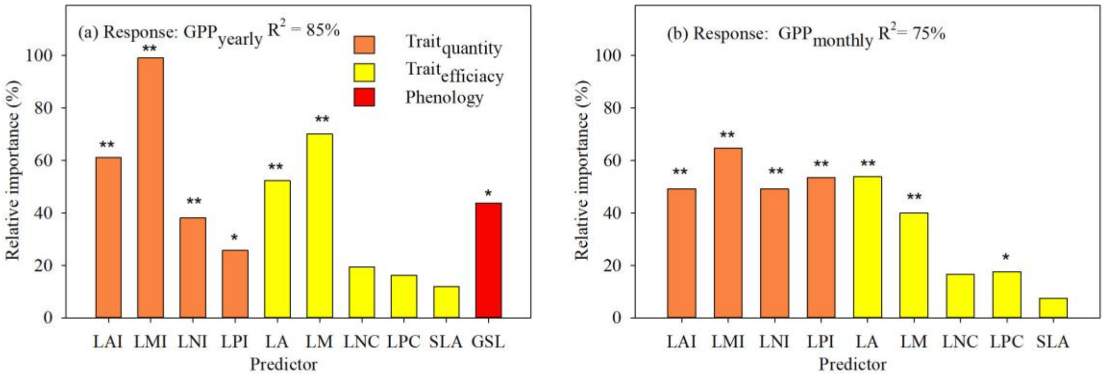

Figure S1 Relative importance of different trait variables from random forest models: (a) annual gross primary productivity (GPP_{yearly}) and (b) monthly gross primary productivity ($GPP_{monthly}$); LA, leaf area (cm^2); LM, leaf dry mass (g); SLA, specific leaf area (cm^2/g); LNC, leaf nitrogen concentration (mg/g); LPC, leaf phosphorus concentration (mg/g); LAI, leaf area index (m^2/m^2); LMI, leaf mass index (g/m^2); LNI, total leaf nitrogen per unit land area (g/m^2); LPI, total leaf phosphorus per unit land area (g/m^2); $*P < 0.05$, $**P < 0.01$.

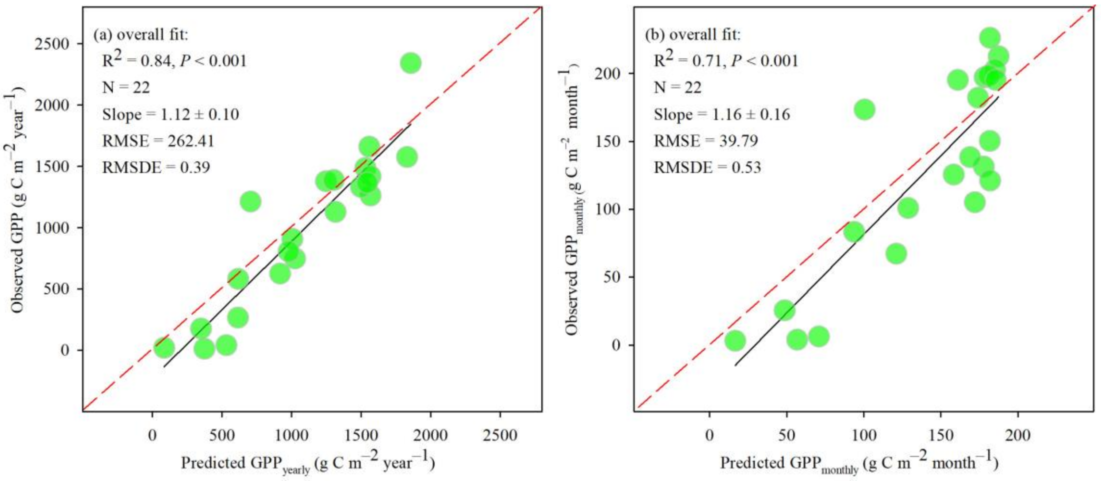

Figure S2 Observed gross primary productivity (GPP) versus predicted GPP for yearly (a) and monthly (b). Predicted productivity of 30% (n = 22) randomly selected sites based on a random forest model obtained using the other 70% (n = 50) of the sites. The red dotted line represents the 1:1 line. RMSE, root mean square error; RMSDE, standardized root mean square error.

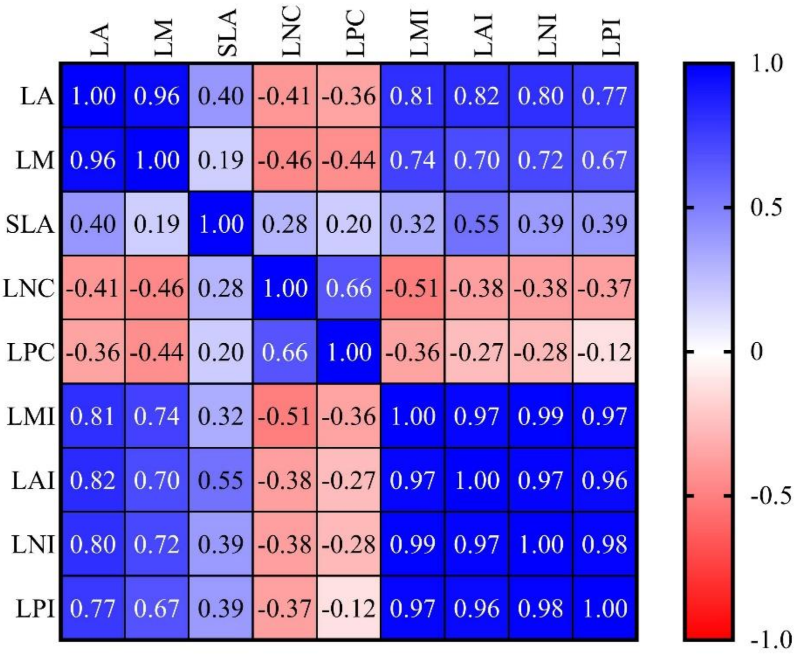

Figure S3 Correlation matrix among traits. LA, leaf area (cm^2); LM, leaf dry mass (g); SLA, specific leaf area (cm^2/g); LNC, leaf nitrogen concentration (mg/g); LPC, leaf phosphorus concentration (mg/g); LAI, leaf area index (m^2/m^2); LMI, leaf mass index (g/m^2); LNI, total leaf nitrogen per unit land area (g/m^2); LPI, total leaf phosphorus per unit land area (g/m^2).

9) References:

1 Luo, T. X. *Patterns of Biological Production and its Mathematical Models for Main Forest Types of China* PhD thesis, Chinese Academy of Sciences, (1996).

2 Luo, T. X., Wang, X. K. & L, F. *Comprehensive Database of Biomass Regressions for China's Tree Species*. (China Forestry Publishing House, 2015).

3 Xie, Z. Q., Wang, Y., Tang, Z. Y. & Xu, W. T. *Handbook of biomass models for common shrubs in China*. (Longmen publishing company, 2018).

4 Niu, S., Classen, A. T. & Luo, Y. Functional traits along a transect. *Functional Ecology* **32**, 4-9, doi:https://doi.org/10.1111/1365-2435.13023 (2018).

5 Zhang, J. *et al.* C: N: P stoichiometry in China's forests: From organs to ecosystems. *Functional Ecology* **32**, 50-60 (2018).

6 Li, Q. *et al.* Regional response of grassland productivity to changing environment conditions influenced by limiting factors. *PLOS ONE* **15**, e0240238, doi:10.1371/journal.pone.0240238 (2020).

7 Xu, L. *et al.* Local community assembly processes shape β-diversity of soil phoD-harbouring communities in the Northern Hemisphere steppes. *Global Ecology and Biogeography* **n/a**, doi:https://doi.org/10.1111/geb.13385 (2021).

8 Liu, Z. *et al.* Divergent long- and short-term responses to environmental gradients in specific leaf area of grassland species. *Ecological Indicators* **130**, 108058, doi:https://doi.org/10.1016/j.ecolind.2021.108058 (2021).

9 Zhao, N. *et al.* Coordinated pattern of multi‐element variability in leaves and roots across C hinese forest biomes. *Global Ecology and Biogeography* **25**, 359-367 (2016).

10 Zhang, J. *et al.* Allocation strategies for nitrogen and phosphorus in forest plants. *Oikos* **127**, 1506-1514, doi:10.1111/oik.05517 (2018).

11 He, N. *et al.* Ecosystem Traits Linking Functional Traits to Macroecology. *Trends in Ecology & Evolution* **34**, 200-210, doi:https://doi.org/10.1016/j.tree.2018.11.004 (2019).

12 Zhao, N. *et al.* Conservative allocation strategy of multiple nutrients among major plant organs: From species to community. *Journal of Ecology* **108**, 267-278, doi:10.1111/1365-2745.13256 (2020).

13 Hilty, J., Muller, B., Pantin, F. & Leuzinger, S. Plant growth: the What, the How, and the Why. *New Phytologist* **232**, 25-41, doi:https://doi.org/10.1111/nph.17610 (2021).

14 Xia, J. *et al.* Joint control of terrestrial gross primary productivity by plant phenology and physiology. *Proceedings of the National Academy of Sciences* **112**, 2788-2793 (2015).

15 Chapin III, F. S. Effects of plant traits on ecosystem and regional processes: a conceptual framework for predicting the consequences of global change. *Annals of Botany* **91**, 455-463 (2003).

16 Garnier, E. *et al.* Plant functional markers capture ecosystem properties during secondary succession. *Ecology* **85**, 2630-2637 (2004).

17 Wright, I. J. *et al.* Global climatic drivers of leaf size. *Science* **357**, 917-921 (2017).
18 Bruelheide, H. *et al.* Global trait–environment relationships of plant communities. *Nature Ecology & Evolution* **2**, 1906-1917, doi:10.1038/s41559-018-0699-8 (2018).
19 Wieczynski, D. J. *et al.* Climate shapes and shifts functional biodiversity in forests worldwide. *Proceedings of the National Academy of Sciences* **116**, 587-592 (2019).
20 Enquist, B. J. *et al.* in *Advances in Ecological Research* Vol. 52 (eds Samraat Pawar, Guy Woodward, & Anthony I. Dell) 249-318 (Academic Press, 2015).

Supplementary Figure 2 Trace plots and posterior density plots of the Bayesian piecewise structural equation modelling with annual GPP. The left panel represents posterior distribution plots for the model parameters and the right panel represents the trace plots showing convergence of the chains. The acronyms Tcpc1, Tepc1 and Tepc2 in turn indicate $Trait_{quantity\ pc1}$, $Trait_{efficiency\ pc1}$ and $Trait_{efficiency\ pc2}$.

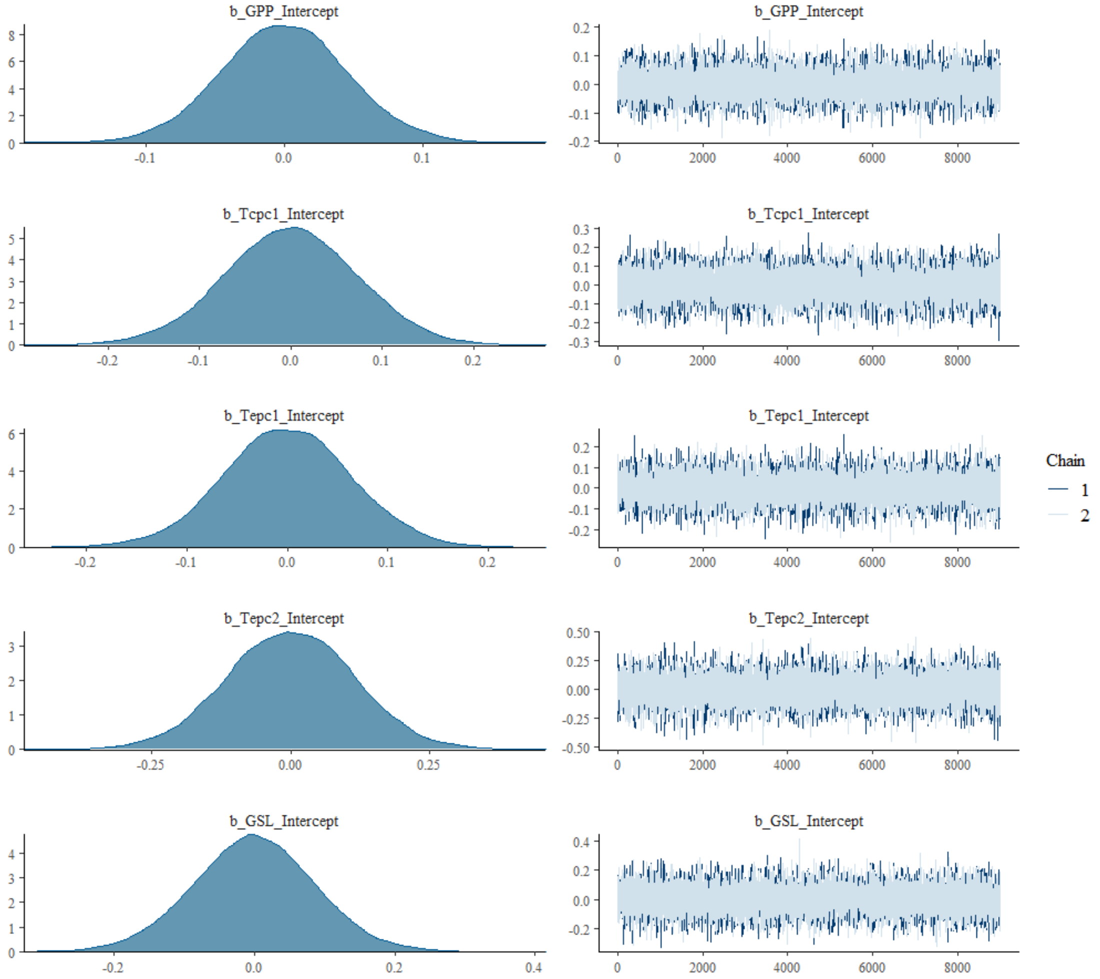

(continued)

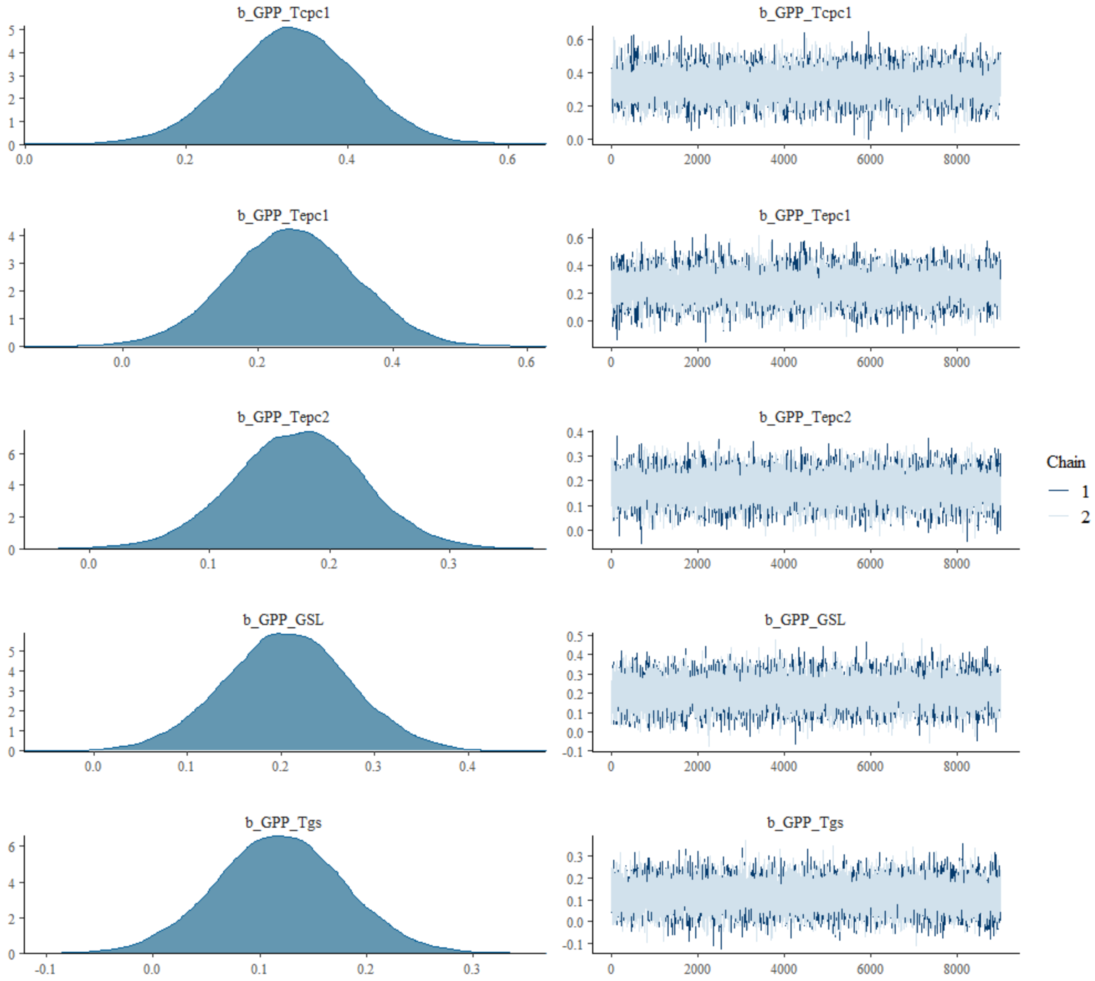

(continued)

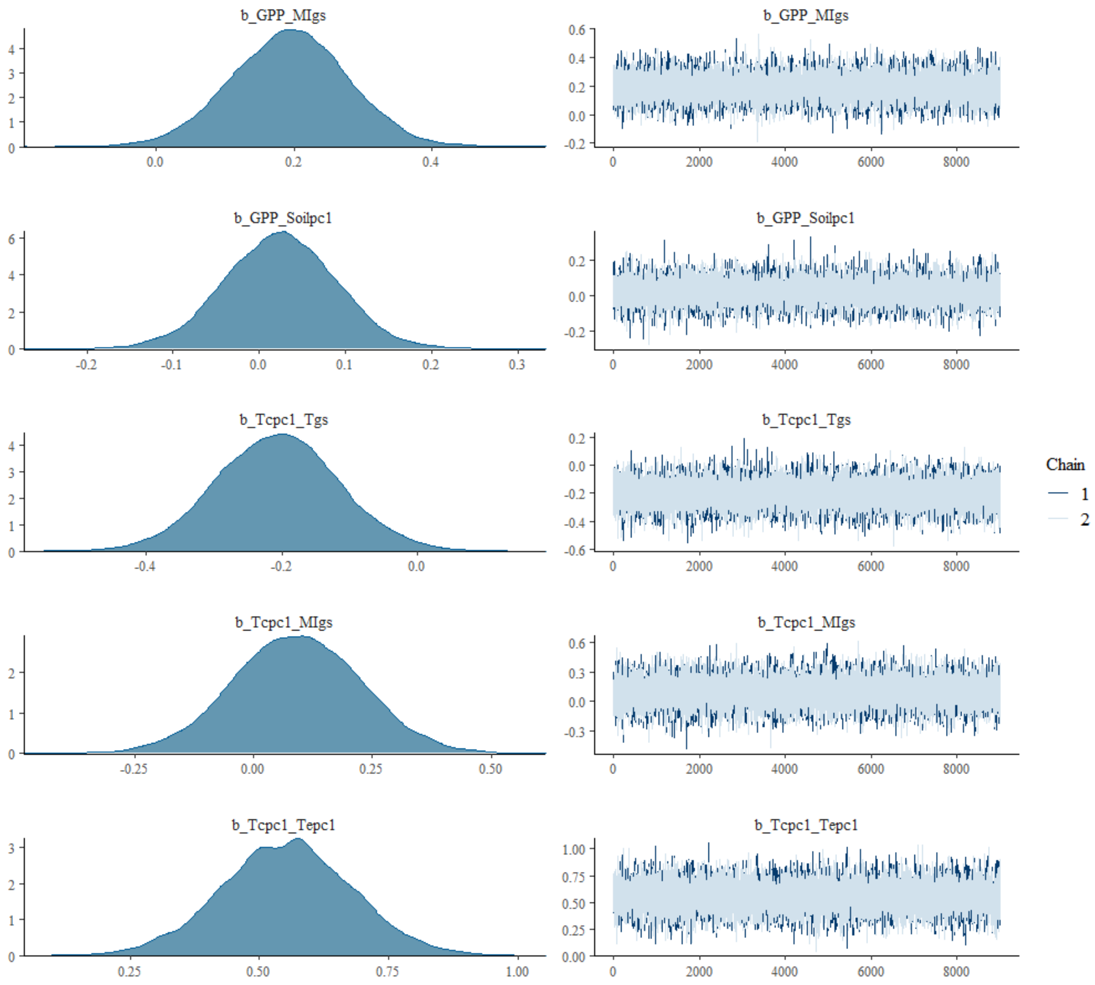

b_GPP_MIgs
b_GPP_MIgs
b_GPP_Soilpc1
b_GPP_Soilpc1
b_Tcpc1_Tgs
b_Tcpc1_Tgs
b_Tcpc1_MIgs
b_Tcpc1_MIgs
b_Tcpc1_Tepc1
b_Tcpc1_Tepc1
Chain
1
2
0
2000
4000
6000
8000

(continued)

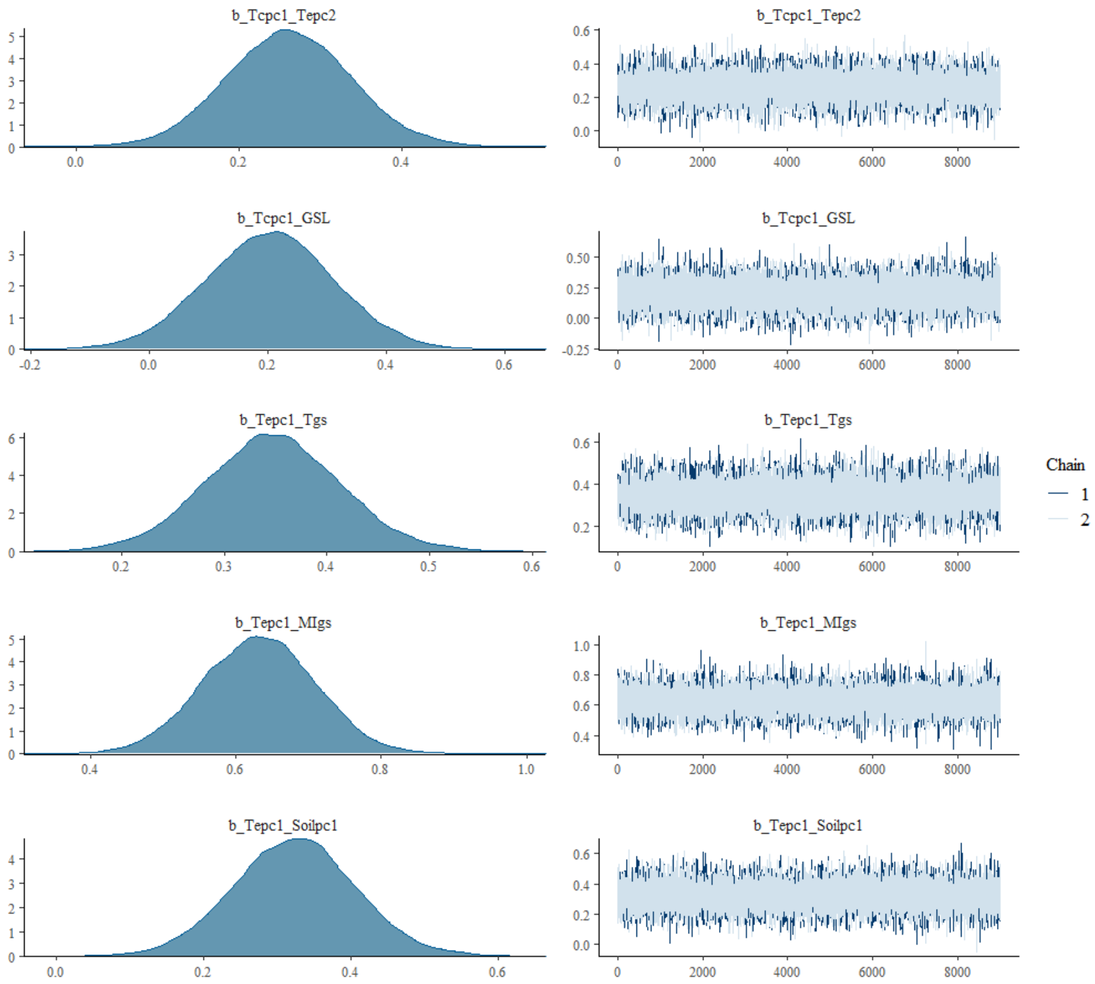
b_Tcpc1_Tepc2
b_Tcpc1_GSL
b_Tepc1_Tgs
b_Tepc1_MIgs
b_Tepc1_Soilpc1
Chain
1
2

(continued)

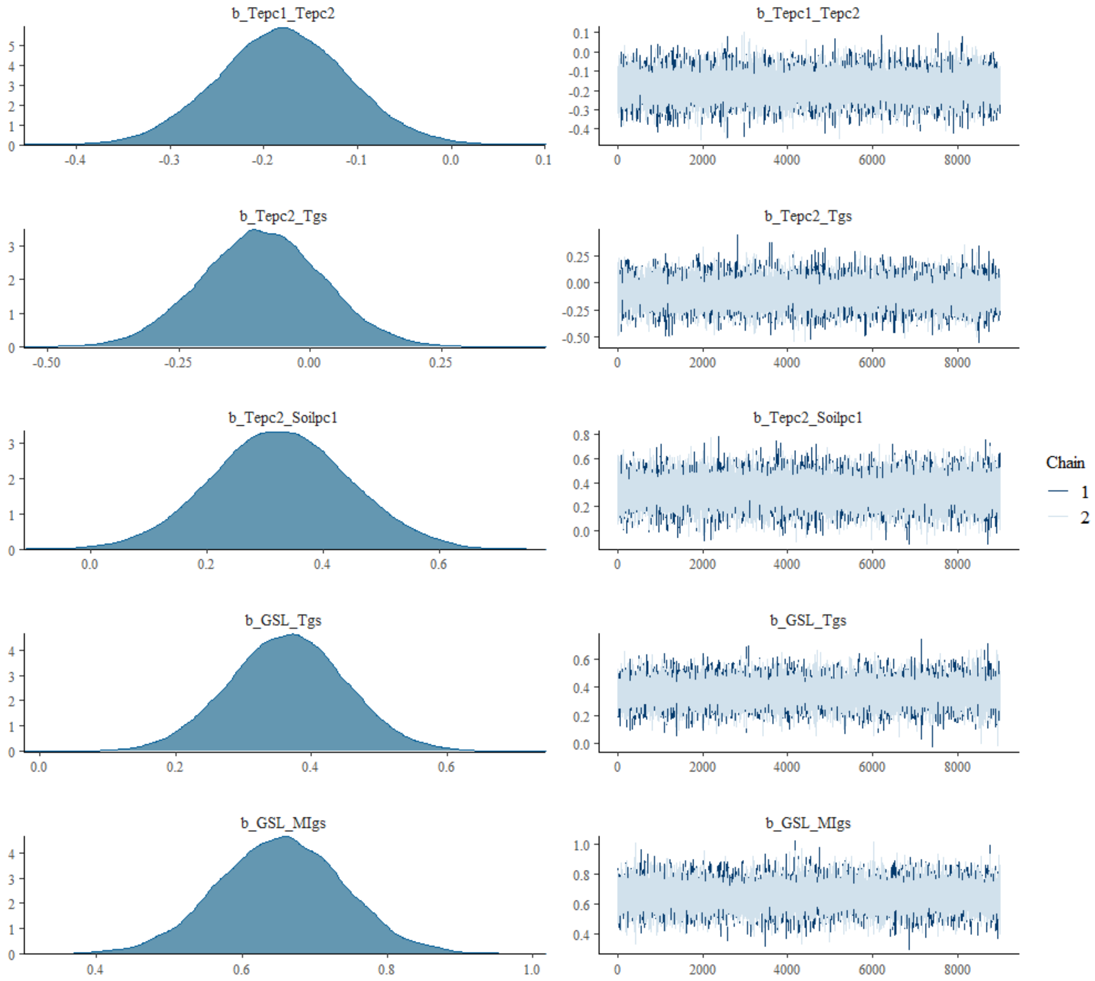

b_Tepc1_Tepc2
b_Tepc2_Tgs
b_Tepc2_Soilpc1
b_GSL_Tgs
b_GSL_MIgs
Chain
1
2

(continued)

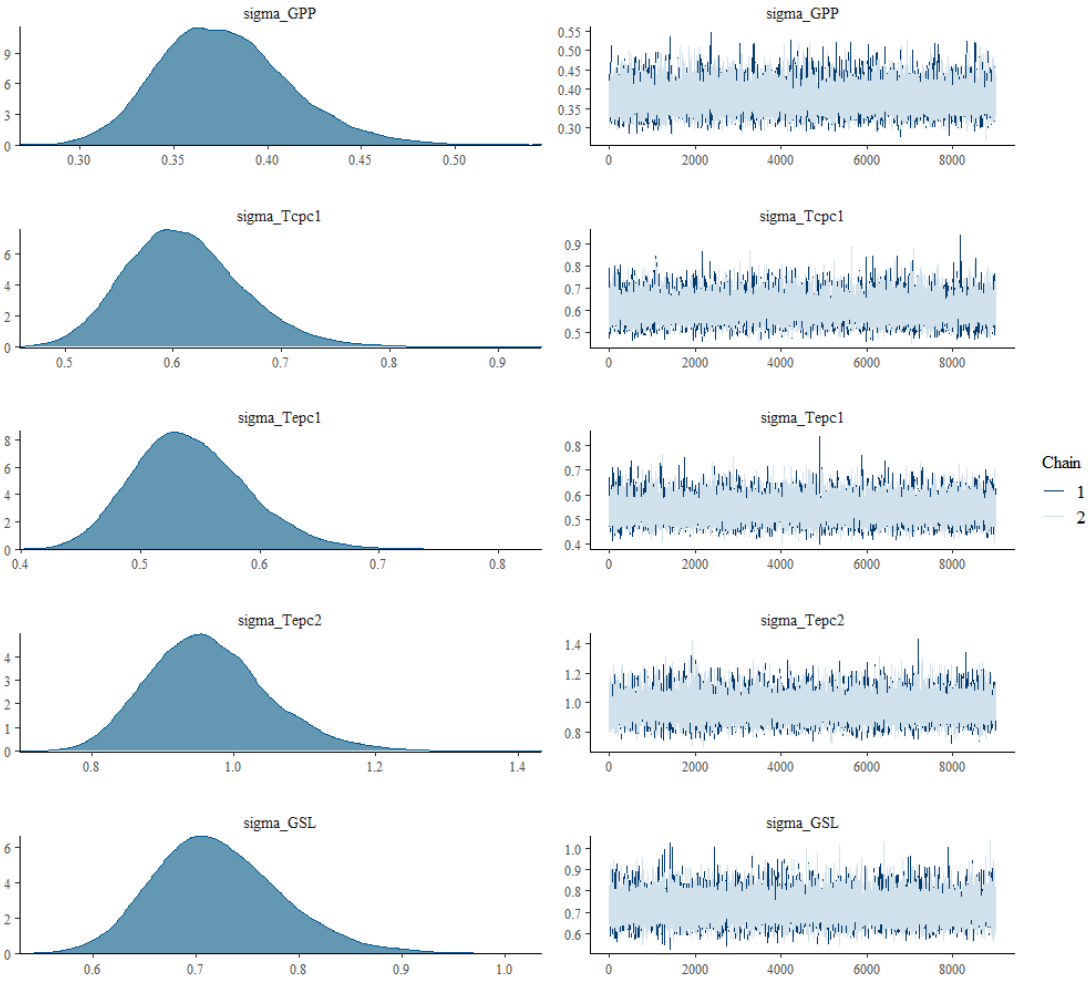
sigma_GPP
sigma_Tcpc1
sigma_Tepc1
sigma_Tepc2
sigma_GSL
Chain
1
2

Supplementary Figure 3 Posterior predictive checks of the mean of the Y's of the Bayesian Structural Equation Model with annually GPP. The histogram represents the posterior distribution and the vertical line represents the mean of endogenous variables (GPP, GSL, $\text{Trait}_{\text{quantity pc1}}$, $\text{Trait}_{\text{efficiency pc1}}$ and $\text{Trait}_{\text{efficiency pc2}}$, in that order from top to bottom.).

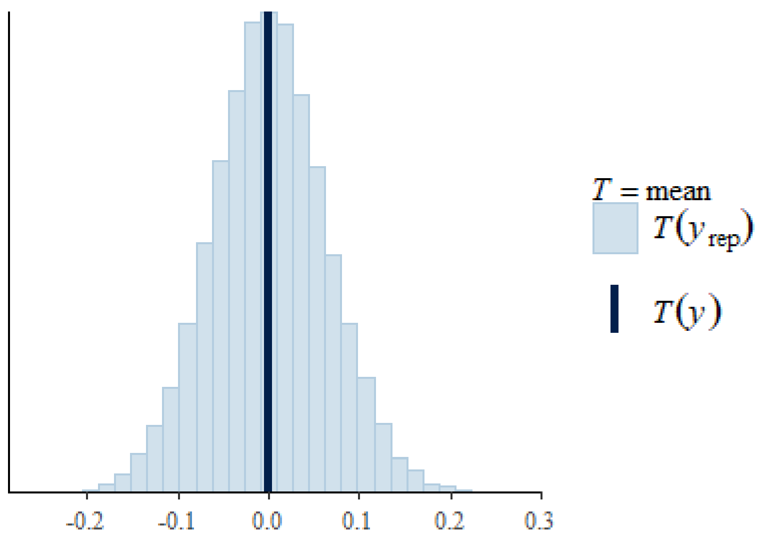

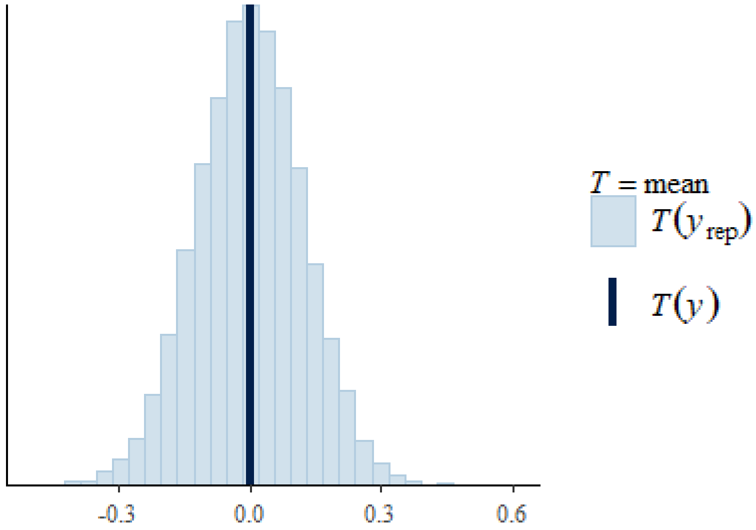
-0.3
0.0
0.3
0.6
T = mean
$T(y_{\text{rep}})$
$T(y)$

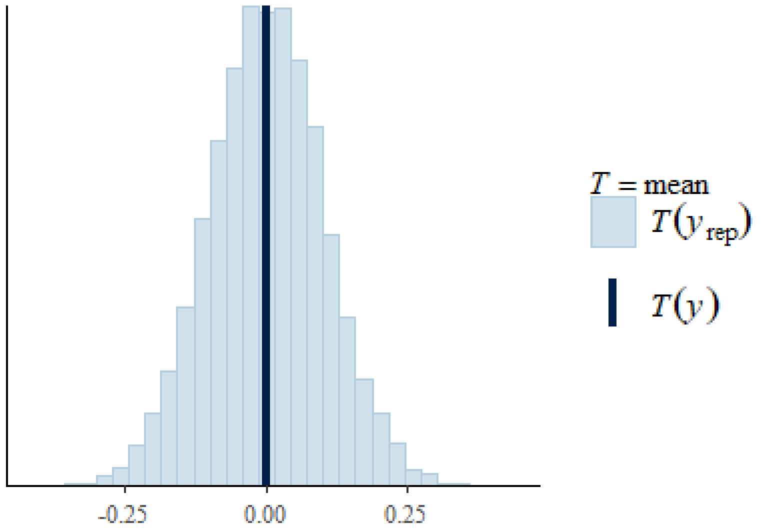

-0.25
0.00
0.25
T = mean
$T(y_{\text{rep}})$
$T(y)$

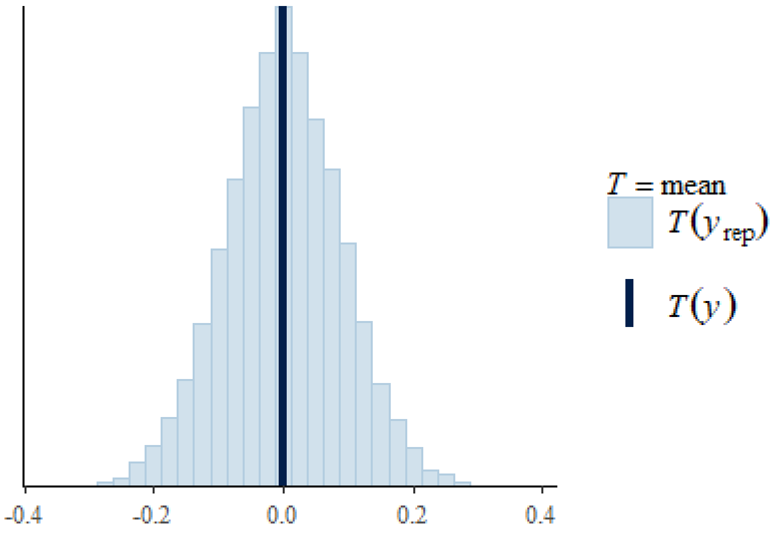

-0.4
-0.2
0.0
0.2
0.4
T = mean
T(y_rep)
T(y)

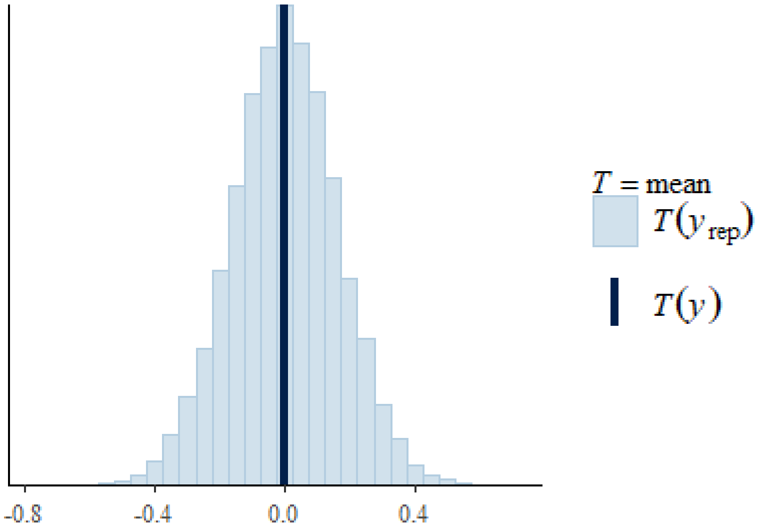

-0.8
-0.4
0.0
0.4
T = mean
T(y_rep)
T(y)

Supplementary Figure 4 Trace plots and posterior density plots of the Bayesian piecewise structural equation modelling with monthly GPP (GPP/GSL). The left panel represents posterior distribution plots for the model parameters and the right panel represents the trace plots showing convergence of the chains. The acronyms Tcpc1, Tepc1 and Tepc2 in turn indicate $\text{Trait}_{\text{quantity pc1}}$, $\text{Trait}_{\text{efficiency pc1}}$ and $\text{Trait}_{\text{efficiency pc2}}$.

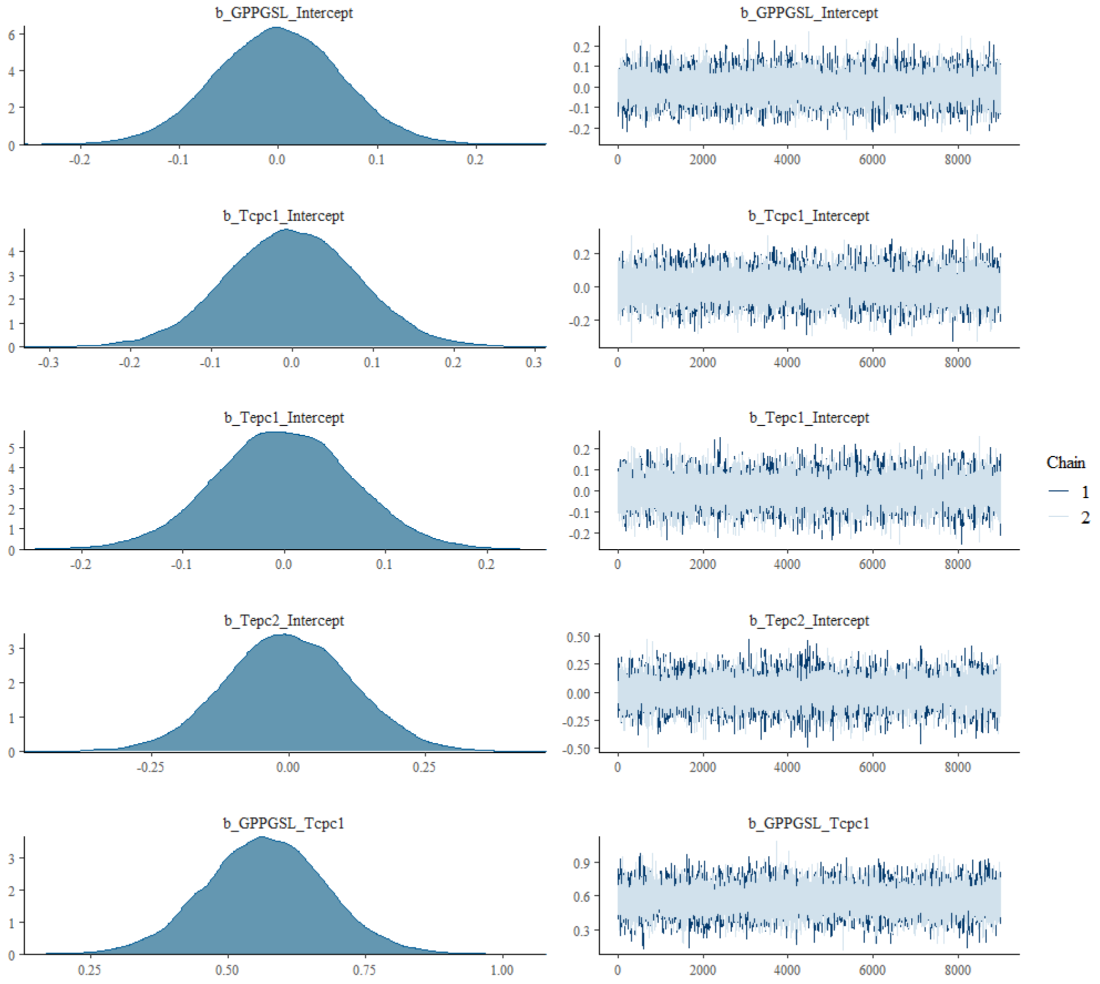

(continued)

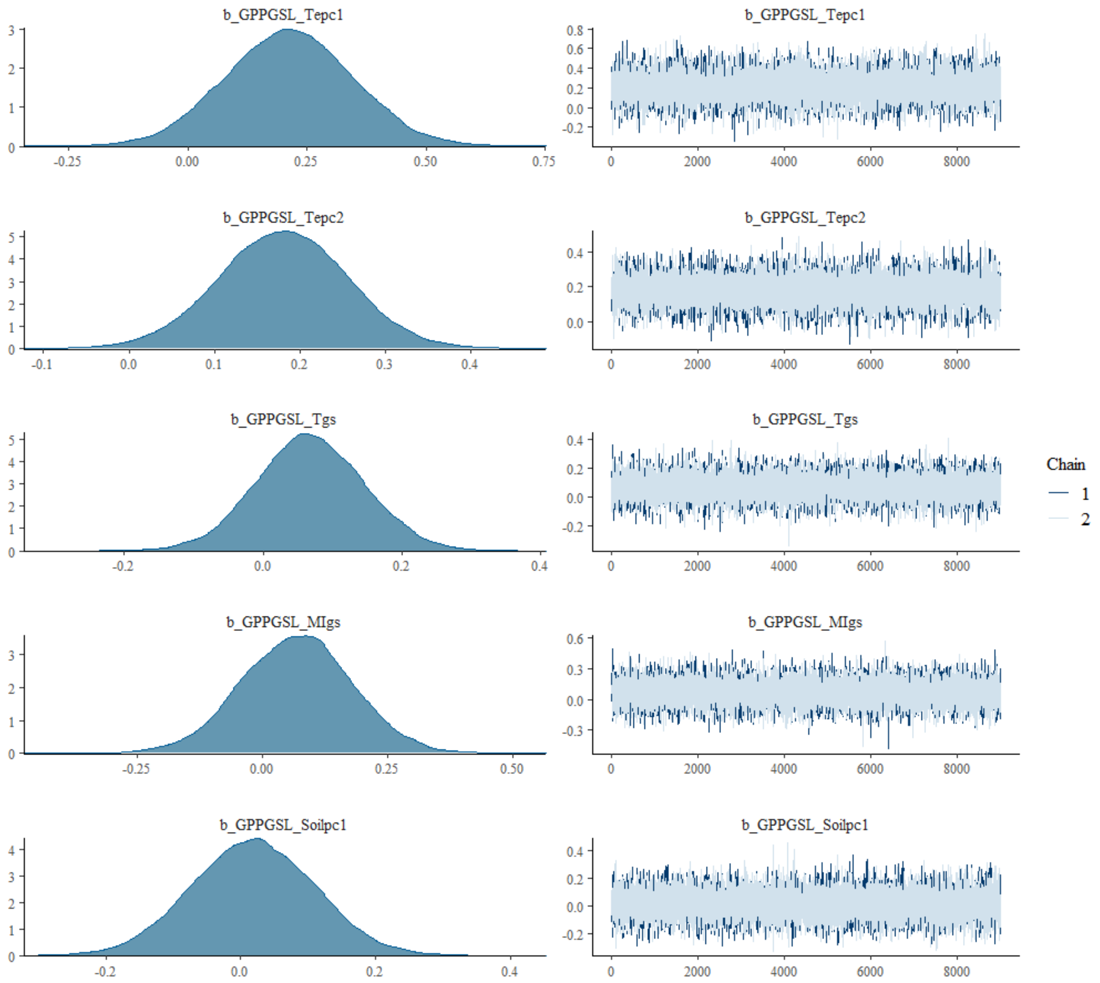

b_GPPGSL_Tepc1
b_GPPGSL_Tepc2
b_GPPGSL_Tgs
b_GPPGSL_MIgs
b_GPPGSL_Soilpc1
Chain
1
2

(continued)

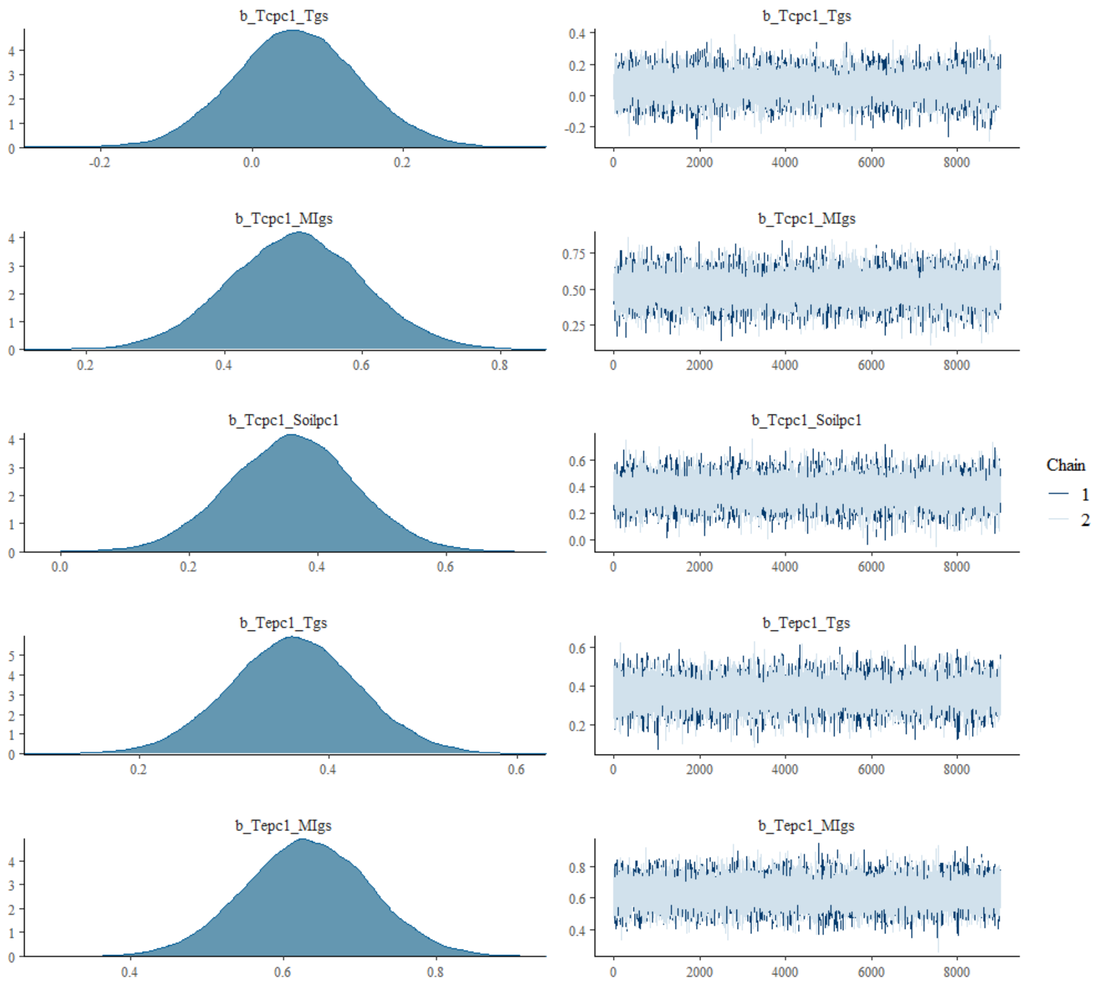
b_Tcpc1_Tgs
b_Tcpc1_MIgs
b_Tcpc1_Soilpc1
b_Tepc1_Tgs
b_Tepc1_MIgs
Chain
1
2

(continued)

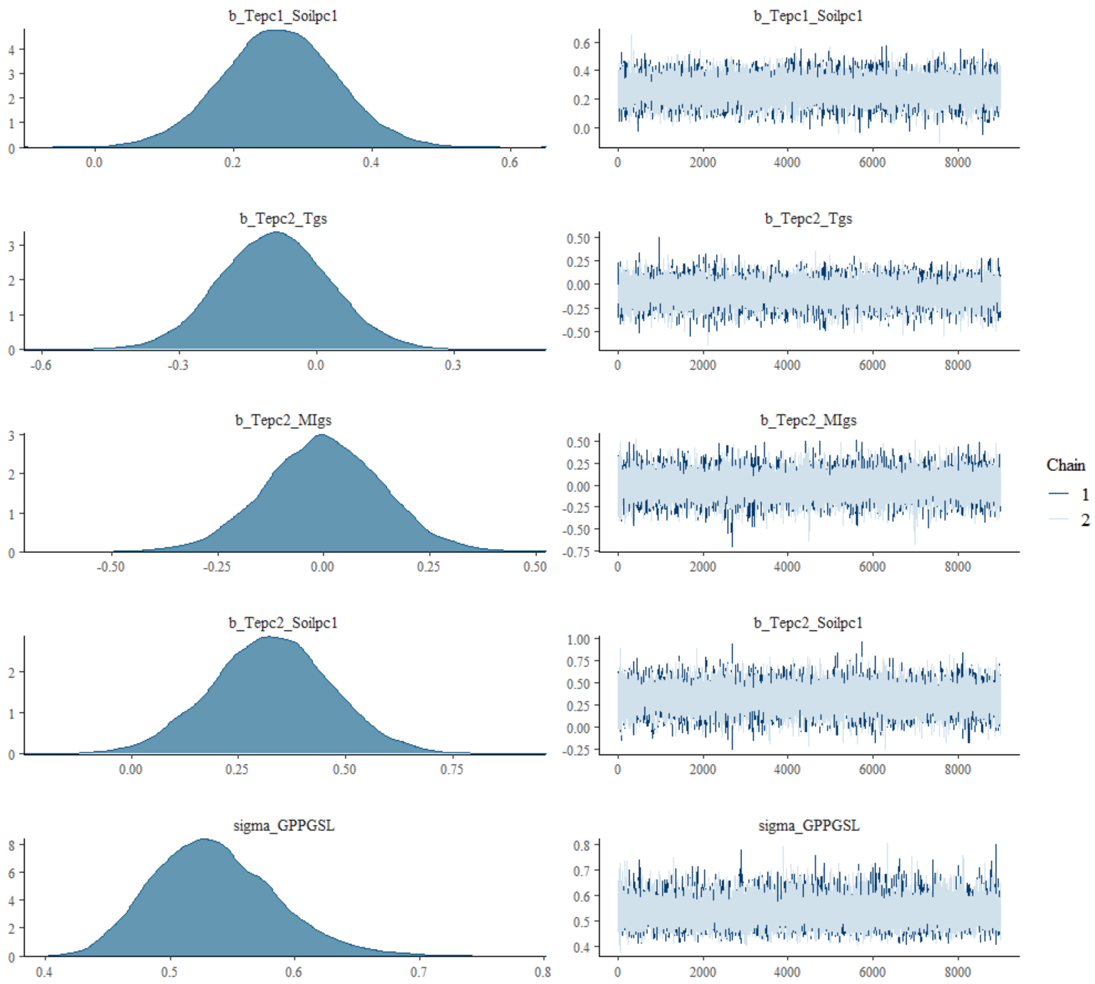
b_Tepc1_Soilpc1
b_Tepc2_Tgs
b_Tepc2_MIgs
b_Tepc2_Soilpc1
sigma_GPPGSL
Chain
1
2

(continued)

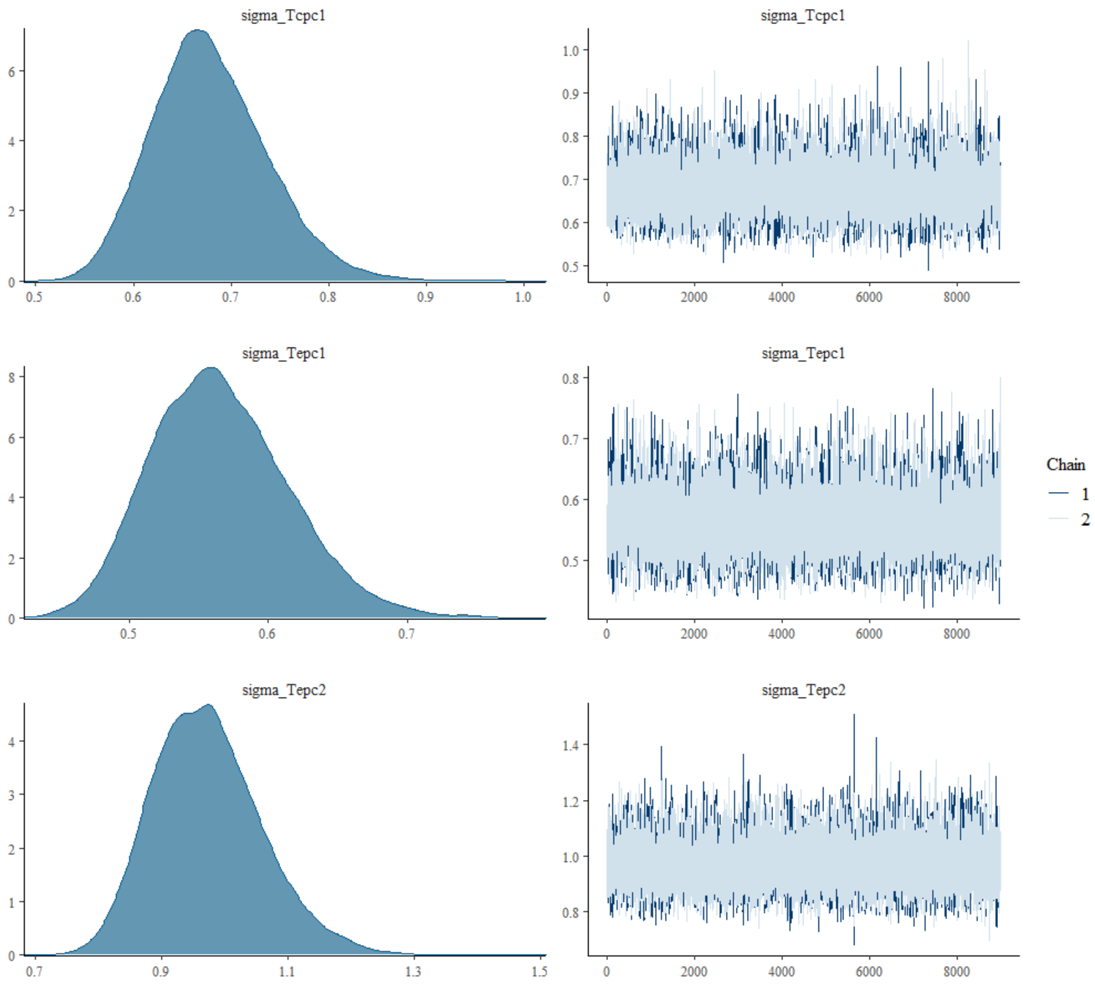
sigma_Tcpc1
sigma_Tcpc1
sigma_Tepc1
sigma_Tepc1
sigma_Tepc2
sigma_Tepc2
Chain
1
2

Supplementary Figure 5 Posterior predictive checks of the mean of the Y's of the Bayesian Structural Equation Model with monthly GPP (GPP/GSL**).** The histogram represents the posterior distribution and the vertical line represents the mean of endogenous variables (GPP/GSL, $\text{Trait}_{\text{quantity pc1}}$, $\text{Trait}_{\text{efficiency pc1}}$ and $\text{Trait}_{\text{efficiency pc2}}$, in that order from top to bottom).

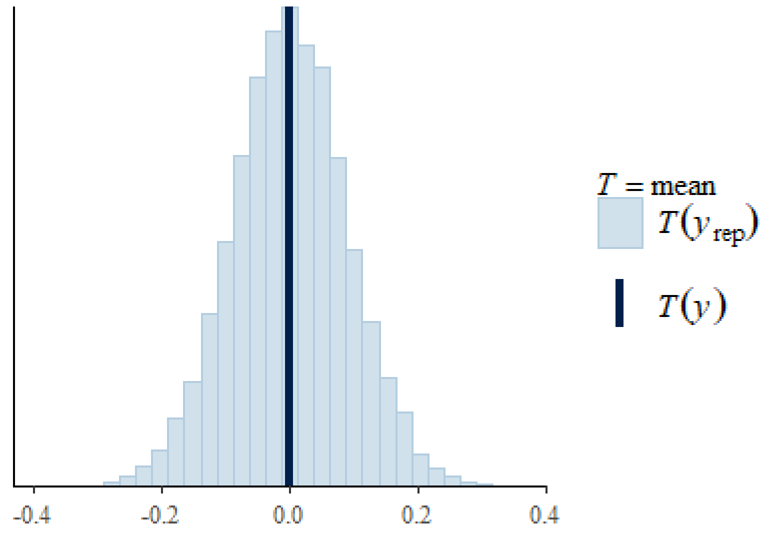

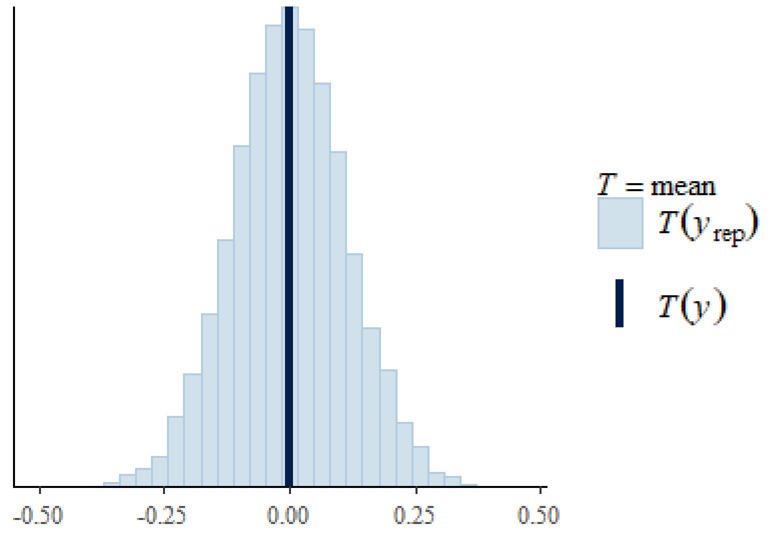
-0.50
-0.25
0.00
0.25
0.50
T = mean
$T(y_{\text{rep}})$
$T(y)$

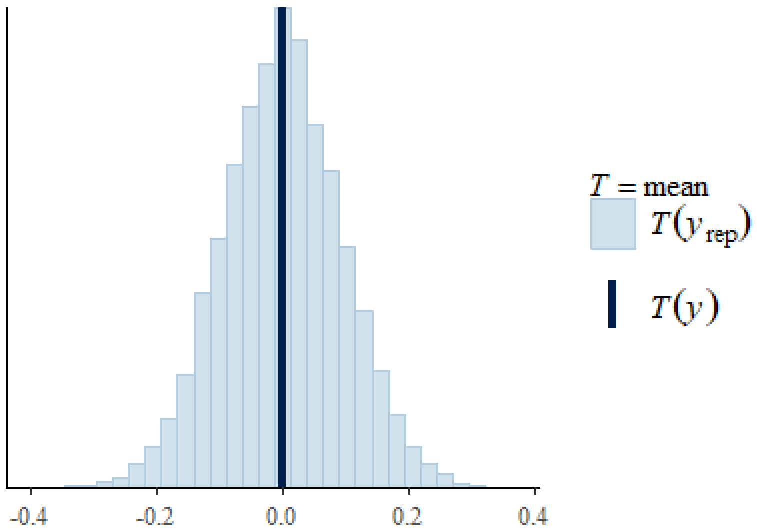
-0.4
-0.2
0.0
0.2
0.4
T = mean
$T(y_{\text{rep}})$
$T(y)$

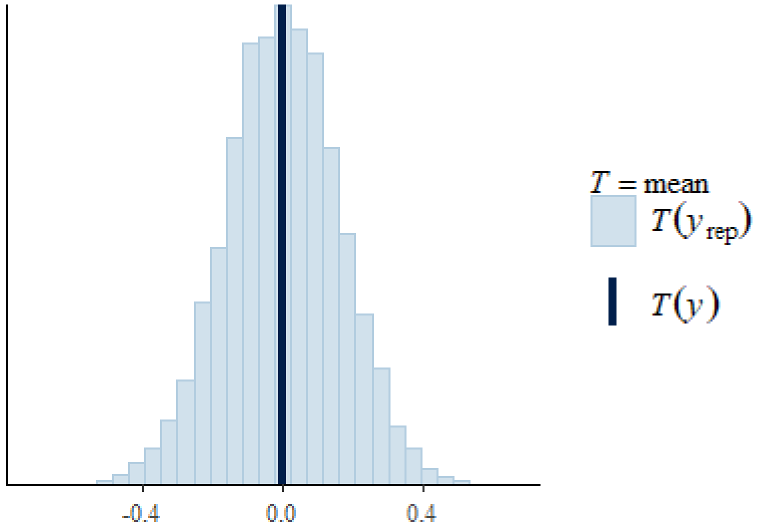

-0.4
0.0
0.4
T = mean
T(y_rep)
T(y)
