# Supplementary material for: Integrating multiple plant functional traits to predict ecosystem productivity
Source: Commun Biol. 2023 Mar 3;6:239. doi: 10.1038/s42003-023-04626-3 (PMC9984401; doi:10.1038/s42003-023-04626-3)
Supplement: Supplementary file 3 — Description of Additional Supplementary Data [file 42003_2023_4626_MOESM3_ESM.docx]

**Description of Additional Supplementary Files**

**File name:** Supplementary Data 1

**Description:** The source data behind the graphs in the paper (i.e., Bayesian piecewise structural equation modelling statistical results for yearly GPP).

**File name:** Supplementary Data 2

**Description:** The source data behind the graphs in the paper (i.e., Bayesian piecewise structural equation modelling statistical results for yearly GPP from individual traits)
